# Characterization of two putative *Dichelobacter nodosus* footrot vaccine antigens identifies the first lysozyme inhibitor in the genus

**DOI:** 10.1038/s41598-019-46506-z

**Published:** 2019-07-11

**Authors:** Maria Victoria Humbert, Alexandra Jackson, Christian M. Orr, Ivo Tews, Myron Christodoulides

**Affiliations:** 1 Neisseria Research Group, Molecular Microbiology, Academic Unit of Clinical and Experimental Sciences, Sir Henry Wellcome Laboratories, University of Southampton Faculty of Medicine, Southampton, United Kingdom; 2 Beamline I23, Diamond Light Source, Harwell Science and Innovation Campus, Didcot, Oxfordshire United Kingdom; 3 Biological Sciences, Institute for Life Sciences, B85 Highfield Campus, University of Southampton, Southampton, United Kingdom

**Keywords:** Protein vaccines, Vaccines

## Abstract

The Gram-negative anaerobic bacterium *Dichelobacter nodosus* (Dn) causes footrot in ruminants, a debilitating and highly contagious disease that results in necrotic hooves and significant economic losses in agriculture. Vaccination with crude whole-cell vaccine mixed with multiple recombinant fimbrial proteins can provide protection during species-specific outbreaks, but subunit vaccines containing broadly cross-protective antigens are desirable. We have investigated two *D. nodosus* candidate vaccine antigens. Macrophage Infectivity Potentiator Dn-MIP (DNO_0012, DNO_RS00050) and Adhesin Complex Protein Dn-ACP (DNO_0725, DNO_RS06795) are highly conserved amongst ~170 *D. nodosus* isolates in the https://pubmlst.org/dnodosus/ database. We describe the presence of two homologous ACP domains in Dn-ACP with potent C-type lysozyme inhibitor function, and homology of Dn-MIP to other putative cell-surface and membrane-anchored MIP virulence factors. Immunization of mice with recombinant proteins with a variety of adjuvants induced antibodies that recognised both proteins in *D. nodosus*. Notably, immunization with fimbrial-whole-cell Footvax vaccine induced anti-Dn-ACP and anti-Dn-MIP antibodies. Although all adjuvants induced high titre antibody responses, only antisera to rDn-ACP-QuilA and rDn-ACP-Al(OH)_3_ significantly prevented rDn-ACP protein from inhibiting lysozyme activity *in vitro*. Therefore, a vaccine incorporating rDn-ACP in particular could contribute to protection by enabling normal innate immune lysozyme function to aid bacterial clearance.

## Introduction

*Dichelobacter nodosus* (formerly *Bacteroides nodosus* and *Fusiformis nodosus*) is a rod shaped, gram negative, obligate anaerobe, non-spore forming bacterium that causes footrot in ruminants^1,2^. In sheep, footrot is a debilitating, highly contagious necrotic disease that affects hooves, leading to severe economic losses in the wool and meat industries. Beginning as an interdigital dermatitis, the disease progresses to destruction of the epidermal matrix, with consequential necrotic separation of the hoof from the underlying soft tissue^3^. Symptoms are lameness and eventual under-running and detachment of the hoof capsules, animal weakness, weight loss and poor wool growth. *D. nodosus* is not capable of invading healthy hooves on its own and infection is preceded by and accompanied with maceration and colonisation of the interdigital skin by the anaerobe *Fusobacterium necrophorum*^4,5^. Despite their synergistic interaction, *D. nodosus* is considered the primary pathogen. Restricting movement of infected sheep, quarantine of virulent strains, foot bathing in antiseptic solutions and administering broad-spectrum antibiotics are methods used commonly for treatment and disease control.

Vaccination against *D. nodosus* reduces disease prevalence^6^ and monovalent whole-cell^7,8^ and mono-, bi- or multi-valent recombinant fimbrial vaccines^9–11^ can provide protection during footrot outbreaks^12,13^. Fimbriae or pili are recognised as the major protective antigen. Based on fimbrial antigenicity, there are 10 major *D. nodosus* serogroups (A-I and M) and additional heterogeneity has been observed in the form of serotypes^10,14,15^. Monovalent whole cell vaccines are inefficient as they do not confer cross-protection against heterologous serogroups^8^. Monovalent (serotype I) whole cell, multivalent (serogroups A, B1, B2, C-I) recombinant fimbrial vaccine Footvax® is the only vaccine commercially available in endemic countries such as the UK^16,17^. However, Footvax was banned recently in Australia where studies suggested this vaccine was performing poorly^18^, and replaced by commercial Custom Footrot R-Pilus Vaccine® (https://www.dpi.nsw.gov.au/data/assets/pdf_file/0015/711510/Footrot-and-specific-strain-vaccine.pdf), which targets the exact strains of footrot bacteria identified in individual flocks.

Multivalent recombinant fimbrial vaccines can provide protection against homologous serogroups^9–11^. In addition, outbreak-specific vaccination with mono- or bivalent recombinant fimbrial vaccines targeting the disease-causing serogroups has been successful^12,13^. However, if multiple serogroups are present within the same flock, sequentially targeting of all serogroups with mono- or bivalent vaccines is required to overcome disease control failure due to antigenic competition^19,20^. The efficacy of current footrot vaccination programmes is compromised^21^, as i) the vaccines do not confer cross-protection against infection with heterologous serogroups, ii) vaccine efficacy is highly dependent on the adjuvant used^10,22^, iii) simultaneous immunization against multiple antigens is hampered by antigenic competition and iv) sheep immune responses are variable and protection is short-lived. Developing a broadly effective footrot vaccine requires the identification of universal, non-fimbrial antigens that could confer immunity to all serogroups. Myers *et al*. used genome sequencing and *in silico* reverse vaccinology to predict several cross-protective protein antigen candidates^23^. The authors recombinantly expressed 87/99 proteins, annotated by the bioinformatics analyses as surface-exposed or secreted, and used immunoblotting against pooled immune and pre-immune sera from sheep infected with serogroup A strain VCS1001 to test their antigenicity. Eight recombinant proteins reacted with immune but not pre-immune sera. Positives included a FK506 binding protein (FKBP)-type peptidyl propyl cis/trans isomerase (PPIase) Macrophage Infectivity Potentiator (MIP, DNO_0012, DNO_RS00050), which is homologous to other MIP proteins that function as essential cell-surface virulence factors^24,25^ and vaccine antigens^25–28^, and the Adhesin Complex Protein ACP (DNO_0725, DNO_RS06795).

Here, we present a study on i) the antigenicity of monovalent recombinant (r)Dn-MIP and rDn-ACP proteins, using a wide variety of adjuvants, in comparison with commercial Footvax vaccine; ii) describe putative structural features of both proteins based on sequence comparison and structure prediction; iii) characterize Dn-ACP, which has two homologous domains to *Neisseria* spp. lysozyme inhibitor molecules^29,30^, as the first lysozyme inhibitor protein to be reported in *D. nodosus*.

## Results

### Conservation of Dn-ACP and Dn-MIP

DNA sequences of the *dn-acp* gene (DNO_0725/DNO_RS06795) and *dn-mip gene* (DNO_0012/ DNO_RS00050) from *D. nodosus* isolates in the https://pubmlst.org/dnodosus/ database^31^ were translated into amino acid sequences and aligned. The database contains 172 isolates: for *dn-acp*, there were 17 defined alleles that grouped into 4 non-redundant alleles for 170 isolates, with 2 isolates with no allele defined (Supplementary Table 1, Supplementary Fig. 1A). The non-redundant Dn-ACP amino acid sequences were highly conserved and shared >99% identity^32^ (Supplementary Fig. 1B). For *dn-mip* 25 alleles were identified that grouped into 9 non-redundant alleles for 166 isolates; no allele could be defined for 6 isolates (Supplementary Table 2, Supplementary Fig. 2A). Again, the Dn-MIP amino acid sequences were highly conserved, sharing ~97% sequence identity (Supplementary Fig. 2B).

### Structural analyses of *Dichelobacter* ACP

**Figure 1:**
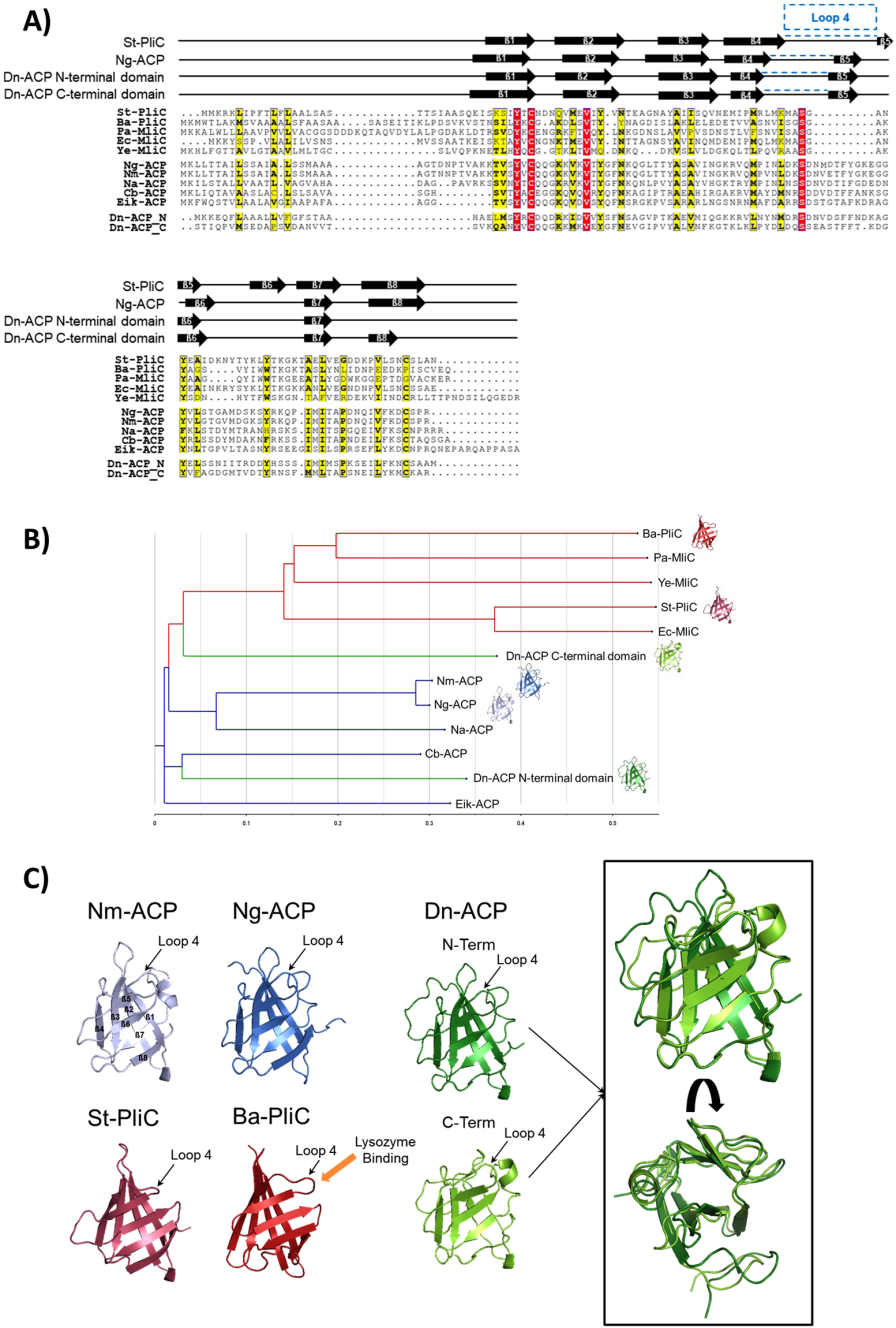
Sequence comparison of Dn-ACP with known bacterial PliC/MliC and ACP proteins. (**A**) Sequence alignment, secondary structure prediction and analysis. The sequences were aligned with ClustalX2 and annotated with secondary structure as predicted by Jpred4 for the two Dn-ACP domains. For comparison, the secondary structure of St-PliC and Ng-ACP is also given (PDBs 3OE3 and 6GQ4 respectively, determined by DSSP). Position of Loop 4 is highlighted by the bracket. Figure prepared using ESPrint. (**B**) Phylogenetic tree. FigTree was used to convert the sequence alignment to a phylogenetic tree. The PliC and MliC proteins are shown as red branches, while the ACP proteins are shown in blue. The Dn-ACP N- and C-terminal domains were aligned and are shown as green branches. Interestingly, the N-terminal ACP domain groups with other ACP proteins, while the C-terminal Dn-ACP domain more closely related to the PliC/MliC family. Ba-PliC (PDB:4ML7), St-PliC (PDB:3OE3), Nm-ACP (PDB:5MY7) and Ng-ACP (PDB:6GQ4) are shown as cartoon next to the respective branch. The reported homology models for Dn-ACP N- and C-terminal domains are also shown. (**C**) Homology modelling of the Dn-ACP N- and C-terminal domains. Structure models for the N- and C-terminal domains of Dn-ACP were predicted using MODELLER and are shown in dark and light green respectively. The boxed overlay highlights subtle structural differences as predicted. For comparison, known structures of Nm-ACP (PDB:5MY7, light blue, with labelled beta strands), Ng-ACP (PDB:6GQ4, blue), St-PliC (PDB:3OE3, pink) and Ba-PliC (PDB:4ML7, dark red) are shown in identical orientation. In all structures, the functional Loop 4 region has been annotated. Additionally, the site of the lysozyme binding interface determined for Ba-PliC has been indicated.

Dn-ACP contains two repetitive segments that both share homology to MliC/PliC and ACP class proteins (Fig. 1A, Table 1). The alignment was done with Clustal software^32^, using the following protein sequences: ACP proteins from *Neisseria meningitidis* (Nm), *Neisseria gonorrhoeae* (Ng), *Neisseria animaloris* (Na), *Comamonadaceae bacterium* (Cb) and *Eikenella corrodens* (Eik); and lysozyme inhibitors or MliC/PliC proteins from *Brucella abortus* (Ba), *Pseudomonas aeruginosa* (Pa), *Yersinia enterocolitica* (Ye), *Salmonella typhimurium* (St) and *Escherichia coli* (Ec). To find the closest relatives, we displayed the Clustal phylogenetic analysis using FigTree, which placed the N-terminal Dn-ACP domain in close relationship with members of the ACP group, while the C-terminal domain is placed between the MliC/PliC and ACP groups (Fig. 1B).

**Table 1:** Sequence identities for ACP and MliC/PliC domains as determined from the sequence alignment shown in Fig. 1.

Pa-MliC	33.1										
St-PliC	20.4	20.9									
Ec-MliC	25.5	19.4	65.2								
Ye-MliC	20.8	25.2	19.6	17							
Nm-ACP	22.2	18.2	18	19.3	23.3						
Ng-ACP	23.1	17.3	18	19.3	23.3	96.8					
Na-ACP	16.7	14.5	17.3	14.8	18.4	50.8	52.5				
Cb-ACP	22.3	18.1	13.5	17.6	14.3	44.8	45.7	42.4			
Ec-ACP	17.1	19.6	15.1	13.5	14.7	38.8	37.9	39.8	41.2		
Dn-ACP-N	16	15.7	18.1	16.5	15.3	34.2	35.1	35.7	42.9	35.4	
Dn-ACP-C	14.1	12.9	14.4	16.7	14.4	36.3	36.3	32.7	36.4	32.4	30.7
	**Ba-PliC**	**Pa-MliC**	**St-PliC**	**Ec-MliC**	**Ye-MliC**	**Nm-ACP**	**Ng-ACP**	**Na-ACP**	**Cb-ACP**	**Ec-ACP**	**Dn-ACP-N**

The ACP and MliC/PliC proteins are overall structurally similar (Fig. 1C blue and red, respectively). However, ACP proteins are distinguished from MliC/PliC proteins by a sequence insertion, occurring after strand β4. Our earlier structure determinations of ACP proteins from the *Neisseria* species Nm-ACP (PDB accession code 5MY7,^29^) and Ng-ACP (PDB accession code, 6GQ4^30^) show this structural feature, annotated as Loop 4 in Fig. 1C. The loop insertion in ACP proteins occurs at a position where the binding interface with lysozyme has been identified for the MliC/PliC proteins^33^. The structural feature of the Loop 4 region allowed us to describe these ACP proteins as novel lysozyme inhibitors^29,30,34^, predicting a different lysozyme interaction mode.

The sequence alignment in Fig. 1A confirms presence of the ACP characteristic sequence insertion in both Dn-ACP domains. The homology model of Dn-ACP domains based on Ng-ACP consequently shows the same feature (green structures in Fig. 1C). The predicted structures for N- and C-terminal Dn-ACP are highly similar (box in Fig. 1C), and both show the Loop 4 insertion, predicting a lysozyme binding mode similar to ACP, but different from MliC/PliC proteins. This allows us to resolve the ambiguity of the phylogenetic prediction, assigning both Dn-ACP domains as *bona fide* ACPs. The predicted structural similarity between Dn-ACP and *Neisseriae* ACP proteins was also supported by the observation that murine antisera to rDn-ACP cross-reacted in Western blotting with rNm-ACP (Supplementary Fig. 3A). Similarly, murine antisera to rNm-ACP^29^ cross-reacted with rDn-ACP protein (Supplementary Fig. 3B).

### Structural analyses of *Dichelobacter* MIP

**Figure 2:**
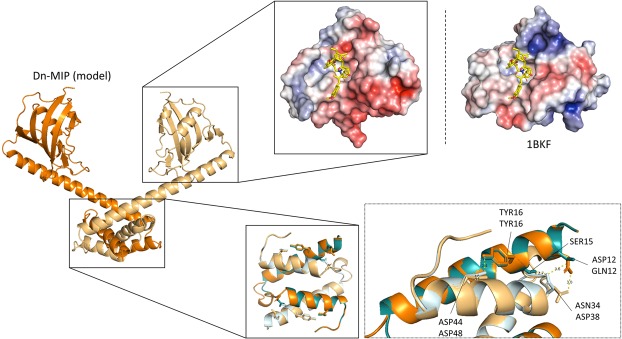
Homology model of Dn-MIP based on the structure of *Legionella pneumophila* Mip (PDB:1FD9). The dimerization domain is shown in the zoom, forming around a hydrophobic core. The dimerisation structure of *Legionella* template (PDB:1FD9, shown in turquoise and light blue) and *D. nodosus* model (shown in gold and orange) are similar overall structure with conserved secondary structure but some changes in the loops connecting secondary structure elements. While the dimer forms around a hydrophobic core, two key polar interactions stabilising the dimer are highlighted. A second zoom shows the catalytic domain in surface presentation with the electric potential mapped onto the surface (blue, positive; red, negative). The position of the inhibitor FK506, taken from the human FKBP protein (PDB:1BKF), indicates presence of the binding pocket in Dn-MIP.

Dn-MIP protein appears to be a classical FKBP-type peptidyl-propyl cis-trans isomerase (PPIase), aligning well with other MIP protein members (Supplementary Fig. 4). A crystal structure is available for the *Legionella pneumophila* (Lp)-MIP protein (PDB: 1FD9)^25,35^. Lp-MIP forms as a V-shaped homo-dimer in the bacterial outer membrane (OM), with the lipidated N-terminal dimerization α-helix anchoring the protein in the OM, and the globular C-terminal domain containing the FKBP-type PPIase function^25,35^. Using the same methods as for Dn-ACP, we have created a homology model for Dn-MIP to gain functional insights into dimerisation and activity (Fig. 2). To understand whether dimerisation would indeed occur, we investigated the dimerisation interface. Using the PISA software, we mapped a very similar interface of nearly 1800 Å^2^ and entropy gain for dimer formation of nearly 23 kcal/mol for template and homology model. Details of the interface are seen in the zoom in Fig. 2. We further investigated conservation of the catalytic domain. Structures of inhibitor complexes to FKBP type proteins are known, *e.g*. the human FKBP protein in complex with the immunosuppressant FK506 (PDB:1BKF), shown in Fig. 2, or the *Trypanosoma cruzi* MIP protein in complex with the same inhibitor (PDB:1JVW). From comparison with these proteins it is clear that while there are changes in structure due to amino acid exchanges, the binding pocket creating the active site is conserved.

### Antigenicity of rDn-MIP and rDn-ACP proteins in different vaccine formulations

Recombinant rDn-ACP encoded by Allele 1 and rDn-MIP encoded by Allele 1 in serotype B strain VPI 2340 [11342] were used in mouse immunisation experiments, using a variety of adjuvant and delivery vehicles that have been shown to enhance the production of antibodies to recombinant proteins: (i) adsorption on Al(OH)_3_ was used as standard, as it is a licensed adjuvant routinely used in veterinary vaccines; (ii) mixing with the saponin Quil A has been used successfully for veterinary applications, including in sheep^36^; (iii) incorporation into liposomes, which provide an intrinsic adjuvant effect and can permit folding of membrane proteins, and are extensively used as a non-toxic delivery vehicle for veterinary vaccine antigens and therapeutic molecules^37–39^; (iv) incorporation into liposomes with additional immunomodulator monophosphoryl lipid A (MPLA)^39^ to increase antigenicity; (v) solubilisation with the zwitterionic detergent Zwittergent (Zw) 3-14, which provides a micellar structure^40^; (vi) solubilisation with Zw 3–14 containing MPLA; (vii) emulsifying in Freund’s water-in oil emulsion and in the (viii) oil emulsion MONTANIDE ISA 71 VG, as used in cattle, poultry and sheep^41,42^, which enable slow antigen release, thus stimulating high and long-lasting antibody responses. Controls included immunisation with Footvax®, adjuvant mixtures without antigen and normal mouse serum (NMS).

**Figure 3:**
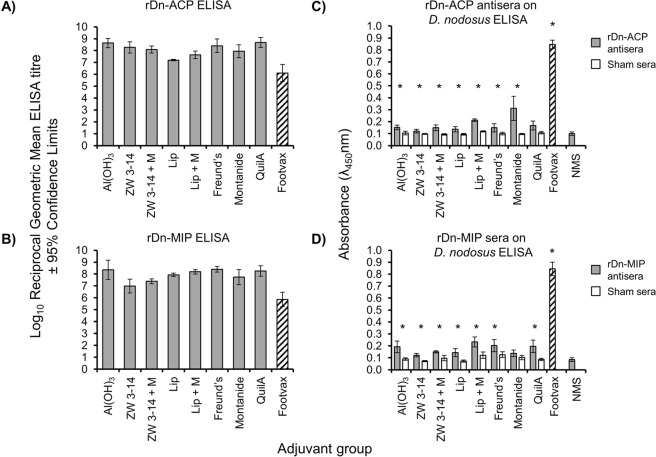
Enzyme-Linked ImmunoSorbent Assay (ELISA) on purified rDn-ACP and rDn-MIP proteins and on *D. nodosus* whole cells (whole-cell ELISA). ELISA reactivity of antisera from individual animals immunised with rDn-ACP or rDn-MIP delivered in Al(OH)_3_, Zwittergent (Zw) 3–14 detergent with or without Monophosphoryl Lipid A (MPLA), liposomes with or without MPLA, Freund’s adjuvant, MONTANIDE and saponin-based vaccine adjuvant Quil-A were reacted against their corresponding immunizing antigens: (**A**) purified rDn-ACP or B) purified rDn-MIP protein. Murine antisera to Footvax was also reacted against both recombinant antigens (**A,B**). The columns represent the geometric mean reciprocal ELISA titres (n = 5 animals per group) and the error bars represent the 95% confidence limits. No significant reactivity was observed with sera from sham-immunised animals or with NMS (absorbance values OD_λ450nm_ < 0.1 for serum dilutions of 1/10; data not shown). All same antisera to (**C**) rDn-ACP or (**D**) rDn-MIP protein were reacted in whole-cell ELISA against *D*. *nodosus* whole cells (1 µg cells/well). The columns represent the average absorbance value (OD_λ450nm_) (n = 5 animals per group) and the error bars represent the standard deviation (SD). Significant reactivity in comparison to the corresponding sham sera or NMS is marked with an asterisk (*) (P < 0.05).

The immune response to rDn-ACP and rDn-MIP proteins was studied initially by the reactivity of individual murine antisera against the homologous protein-ELISA (Fig. 3). There were no significant differences (P > 0.05) between the geometric mean responses of rDn-ACP antisera tested against homologous rDn-ACP (reciprocal end point titres between 16 × 10^6^ and 490 × 10^6^,) (Fig. 3A). Similarly, rDn-MIP antisera tested against homologous rDn-MIP showed no significant differences (P > 0.05) between the adjuvant groups (reciprocal end point titres between 10 × 10^6^ and 245 × 10^6^) (Fig. 3B). Antisera to Footvax reacted with both rDn-ACP and rDn-MIP proteins, with similar reciprocal geometric mean end point titres of ~1 × 10^6^ (Fig. 3A,B). No reactivity was observed with sham-immunized sera or NMS.

Individual antisera were also tested in a *D. nodosus* whole cell ELISA. Serum reactivity was expressed as mean OD λ_450_nm value and standard deviation for each immunization group. All rDn-ACP-adjuvant formulations, except QuilA, elicited similar weak but specific reactivities against *D. nodosus* whole cells, which was statistically significant when compared to the corresponding sham-immunized groups (OD λ_450_nm ~0.2, P < 0.05) (Fig. 3C). All rDn-MIP formulations, except MONTANIDE, elicited weak but specific reactivity (OD_λ450_nm ~0.2) against *D. nodosus* whole cells (Fig. 3D), which were also statistically significant, compared with the corresponding sham-immunized groups (P < 0.05). The mean reactivity of Footvax antisera against whole cells was significantly higher (P < 0.05) compared with any of the recombinant protein formulations, with values of OD_λ450_nm > 0.8. Reactivity with NMS or any of the sham-immunized sera was not significant (P > 0.05) (Fig. 3C,D).

**Figure 4:**
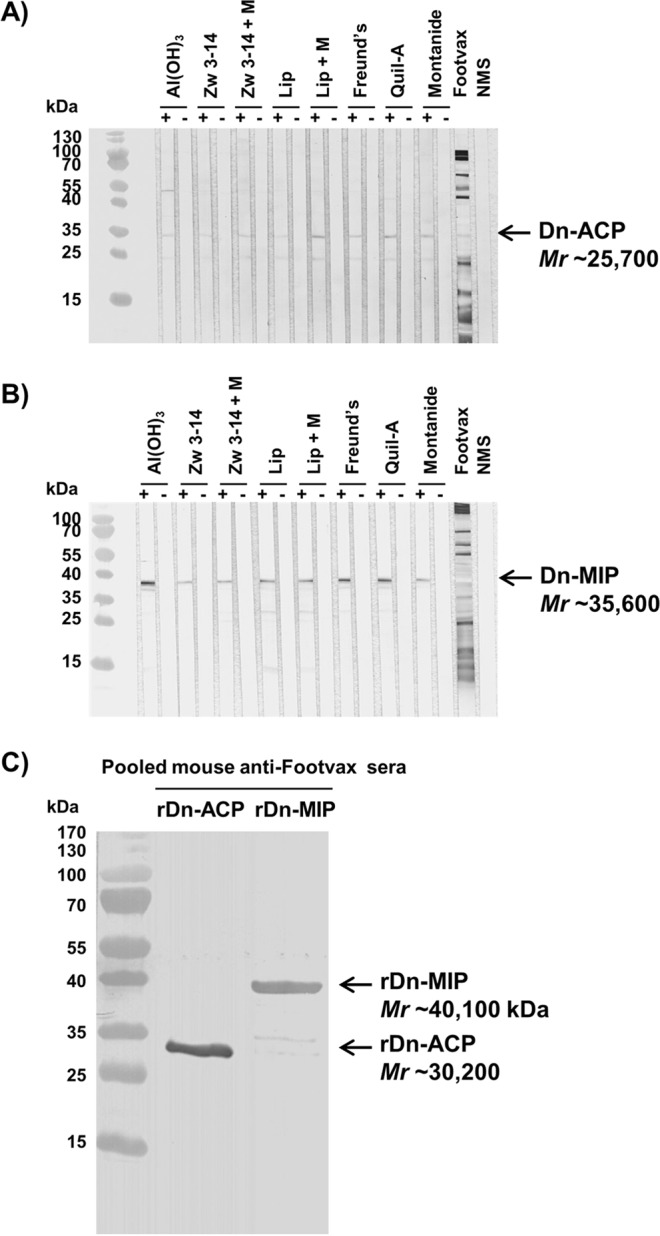
Antigenicity of sera from mice immunized with rDn-ACP or rDn-MIP protein delivered in different vaccine formulations tested by Western blotting. Pooled mice antisera (1/100 dilution; n = 5 animals) raised against purified (**A**) rDn-ACP or B) rDn-MIP protein delivered in Al(OH)_3_, Zwittergent (Zw) 3–14 detergent with or without Monophosphoryl Lipid A (MPLA), liposomes with or without MPLA, Freund’s adjuvant, saponin-based vaccine adjuvant Quil-A and MONTANIDE, as well as antisera to Footvax and normal mouse serum (NMS) as a control, were reacted against wild type *D*. *nodosus* whole cell lysate preparation (50 μg/well or 15 μg/well, respectively) in strip Western blot. Dn-ACP and Dn-MIP proteins were specifically recognised as a single band of *Mr* ~25.7 kDa or ~35.6 kDa, respectively, with all murine antisera to the corresponding single antigen (identified by the arrow). Antisera to Footvax, instead, reacted against multiple proteins in the lysate. All sham immunisation sera and NMS were non-reactive. Samples were tested on strips prepared from the same western blots and processed in parallel. (**C**) Pooled mice antisera (1/100 dilution; n = 5 animals) raised against footrot vaccine Footvax was reacted in Western blot against purified rDn-ACP and rDn-MIP proteins (1 μg each), which were both recognized as a single band of *Mr* ~30.2 kDa or ~40.1 kDa, respectively (identified by the arrow). Samples were tested and processed in parallel. Images were captured on a HP ScanJet flatbed scanner. Representative full-length blots are presented (n ≥ 3 experiments for each western blot).

Testing antisera against whole cell lysates in Western blots showed weak but specific responses against rDn-ACP vaccine formulations (Fig. 4A), and strong and specific immune responses against rDn-MIP vaccine formulations (Fig. 4B). No significant reactivity was observed with any of the respective sham-immunized sera or NMS (Fig. 4A,B). Murine antisera to Footvax reacted strongly against multiple targets in the *D. nodosus* whole cell lysate (Fig. 4A,B) and also recognised both purified recombinant Dn-ACP and Dn-MIP proteins (Fig. 4C).

### Dn-ACP is a lysozyme inhibitor

**Figure 5:**
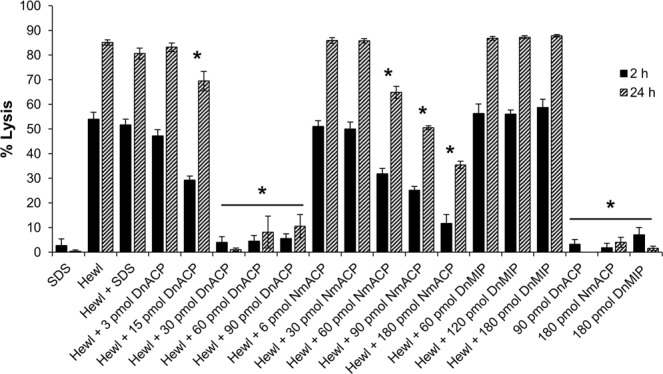
Inhibitory activity of purified rDn-ACP protein on Hen egg-white lysozyme (Hewl) enzymatic function at 2 h and 24 time points *in vitro*. Percentage lysis of *M. lysodeikticus* cell suspension estimated for each condition after 2 h (Supplementary Fig. 5) and 24 h incubation. *M. lysodeikticus* cells alone or in the presence of 90 pmol/well of rDn-ACP, 180 pmol/well of rNm-ACP or 180 pmol/well of rDn-MIP proteins without Hewl were used as negative control conditions for lysis. The positive control sample consisted of *M. lysodeikticus* cells in the presence of Hewl only. The columns represent the mean (for the same n = 3 independent experiments) and the error bars represent the corresponding SEM. Data were compared with a two-sample *t*-Test and the asterisks (*) denote significant difference (P < 0.05) compared to the control of lysis with Hewl only.

The predicted structural similarity of Dn-ACP with *Neisseria* ACP prompted us to test for *in vitro* lysozyme inhibition. Dose-dependent kinetics of purified rDn-ACP inhibition of Hen egg-white lysozyme (Hewl) was determined by measuring lytic absorbance over time of a suspension of *Micrococcus lysodiekticus*, a Gram-positive bacterium that is intrinsically sensitive to lysozyme. Hewl (10 U) induced ~55% lysis of *M. lysodiekticus* by 2 h, which increased to ~85% by 24 h (Fig. 5, Supplementary Fig. 5A). Addition of doses ≤ 3 pmol rDn-ACP was not protective against Hewl lysis (P > 0.05). However, addition of 15 pmol rDn-ACP reduced Hewl-mediated lysis of *M. lysodeikticus* cells by ~45% and ~ 20% after 2 h and 24 h (P < 0.05), respectively (Fig. 5, Supplementary Fig. 5A). Addition of ≥ 30 pmol rDn-ACP reduced Hewl-mediated lysis by ~100% after 2 h (P < 0.05) (Fig. 5, Supplementary Fig. 5A). In comparison, higher amounts of meningococcal rNm-ACP protein were required to produce similar protection, with the addition of 180 pmol rNm-ACP reducing *M. lysodiekticus* lysis by ~80% after 2 h (Supplementary Fig. 5B) and ~59% after 24 h (Fig. 5), compared with the control Hewl. Addition of rDn-MIP did not inhibit Hewl-induced lysis (Fig. 5, Supplementary Fig. 5C), and none of the tested proteins (rDn-ACP, rNm-ACP or rDn-MIP) on their own showed any lytic effect (Fig. 5, Supplementary Fig. 5). The possibility that the trace presence of SDS used to solubilize rDn-ACP and rNm-ACP proteins affected the enzymatic activity of Hewl was also tested. Addition of SDS at the same final concentration in the highest dose of Nm-ACP tested (2.25 × 10^−3^% w/v SDS) to a suspension of the bacteria in the presence or absence of Hewl had no significant effect (P > 0.05) (Fig. 5, Supplementary Fig. 5), confirming that lysis of *M. lysodeikticus* was due to Hewl lytic activity and that inhibition of Hewl in the presence of either rDn-ACP or rNm-ACP was due to the inhibitory activity of these proteins.

Lysozyme can kill bacteria through its hydrolase activity and through an enzyme-independent mechanism that relies on its cation state^43,44^. To exclude the possibility that lysozyme antimicrobial activity was responsible for the observed killing of *M. lysodiekticus*, bacterial suspensions were treated with Hewl that had been boiled for 1 h, a treatment that has been reported to destroy peptidoglycan hydrolase activity, whilst maintaining antimicrobial activity^45,46^. Addition of boiled lysozyme did not induce lysis of *M. lysodiekticus* cells compared to native Hewl (Supplementary Fig. 6), demonstrating that inherent Hewl antimicrobial activity did not induce *M. lysodiekticus* cell death.

### Antibodies to rDn-ACP inhibit Dn-ACP enzymatic activity

**Figure 6:**
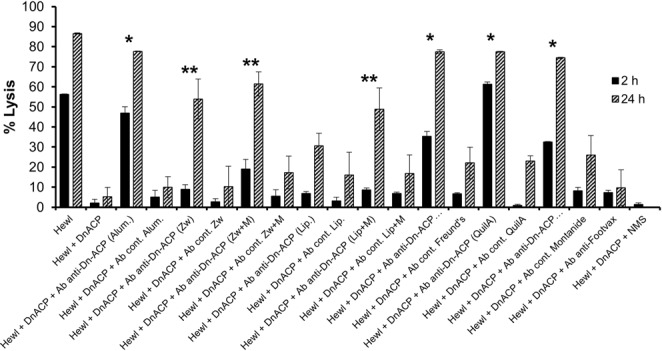
Effect on the levels of restored Hewl lytic activity, initially inhibited by rDn-ACP, influenced by the nature of the adjuvant used to generate antisera to rDn-ACP. Determination of percentage of *M. lysodeikticus* cell lysis for each test condition shown in Supplementary Fig. 7 for 2 h and 24 h incubation time points only. The columns represent the mean % lysis (from n = 3 independent experiments) and the error bars represent the corresponding SEM. Data were compared with a paired *t*-Test and the asterisks denote significant inhibition (P < 0.05) of rDn-ACP function by anti-rDn-ACP sera, compared to treatment without antisera, after 2 h (and continuing after 24 h incubation) (*) or after 24 h only (**).

Antibodies to rNm-ACP and rNg-ACP prevented lysozyme inhibition *in vitro*^29,30^. We tested whether rDn-ACP antisera could restore Hewl lytic activity in the presence of purified rDn-ACP and examined whether this effect was influenced by the adjuvants used. Antisera generated by rDn-ACP-QuilA and rDn-ACP-Al(OH)_3_ prevented Hewl inhibition after 2 hrs by ~100% and 90%, respectively (Fig. 6, Supplementary Fig. 7). Antisera to rDn-ACP-MONTANIDE and rDn-ACP-Freund´s oil-in-water emulsions prevented Hewl inhibition only by ~50–60% (Fig. 6, Supplementary Fig. 7). Inhibition after 24 hrs was comparable in all cases (Fig. 6). By contrast, the inhibitory effect of antisera to rDn-ACP-Zw 3–14 with MPLA was comparatively low after 2 h (~30%), but increased after 24 hrs to ~70% (Fig. 6). Inhibitory effects of antisera to rDn-ACP-Zw 3–14 without MPLA or to rDn-ACP-Liposomes with or without MPLA, was only observed after 24 hrs, and ranged from 30–60% (Fig. 6). As expected, control NMS and all corresponding sham-immunized sera did not prevent rDn-ACP from inhibiting Hewl *in vitro* (Fig. 6). Interestingly, Footvax antisera were unable to prevent rDn-ACP inhibition of Hewl activity (Fig. 6).

## Discussion

The data presented here support proposing rDn-ACP and rDn-MIP antigens as novel antigens in combined vaccine strategies. Based on homology models and structural similarity, we derived structural and functional properties for both proteins. We presented data also on the function of rDn-ACP as a lysozyme inhibitor. Both Dn-ACP and Dn-MIP proteins are highly conserved in the http://pubmlst.org/dnodosus collection of isolates. In our study, we immunized with rDn-ACP encoded by Allele 1 (serotype B strain VPI2340), expressed by 86% of the isolates (146 isolates/170 total), and with rDn-MIP from Allele 1 of the same strain, expressed in 50% of the isolates (83 isolates/166 total). Assuming no cross-reactivity, a monovalent rDn-ACP Allele 1 protein vaccine would cover the majority of isolates, whereas a monovalent rDn-MIP Allele 1 protein vaccine would protect against 50% of disease-causing strains. Given the high sequence identity between non-redundant Dn-ACP and Dn-MIP proteins, levels of coverage might be even higher, assuming protein cross-reactivity.

Modelling of Dn-MIP structure demonstrated similarities with the *Legionella*-MIP, which has been extensively characterised as a FKBP-type PPIase^25^. PPIases are a superfamily of proteins ubiquitously distributed among living organisms, which function primarily to assist the folding and structuring of unfolded and partially folded polypeptide chains and proteins^25^. Lp-MIP and other MIP proteins exist as homo-dimers in the bacterial OM. Each monomer can be structurally divided into three regions: 1) a N-terminal α-helix that participates in a dimerization domain and is probably lipidated to allow anchoring into the OM; 2) a long connecting α-helix in the centre that ends with 3) a globular C-terminal domain containing the FKBP-type PPIase function^25^. MIP-like PPIases have been reported as essential cell-surface and membrane-anchored virulence factors. Lp-MIP was the first genetically identified FKBP-type PPIase that was shown to be required for virulence and intracellular infection^24,47–50^. A *Neisseria gonorrhoeae* MIP homologue was required for intramacrophage survival^51,52^ and a *Neisseria meningitidis* MIP homologue was important for meningococcal survival in the blood^53^. Furthermore, inhibition of Nm-MIP prevented meningococci from adhering, invading and/or surviving in epithelial cells^54^. Several bacteria that cause animal infections, including *Chlamydia, Francisella, Aeromonas* and *Pseudoalteromonas*, amongst many others, also produce MIP-like PPIases essential for virulence^25^. Furthermore, recombinant MIP proteins from *Chlamydia spp*. and *Coxiella burnetti* have been examined as potential vaccine antigens^25^. Based on sequence and structural resemblance and on our previous studies demonstrating that rNm-MIP protein was immunogenic and capable of inducing antibodies bactericidal for meningococci and cross-protective against gonococci^26–28^, we hypothesize that Dn-MIP protein is probably a virulence-associated PPIase and therefore merits investigating as a putative antigen for subunit footrot vaccines.

Sequence analysis revealed duplication of ACP domains in Dn-ACP as a striking observation. We showed that both domains fall structurally into the novel class of lysozyme inhibitors that we identified earlier in the *Neisseriae*^29,30,34^, and these domains had a characteristic loop insertion that alters the lysozyme binding interface. While lysozyme inhibition can be shown for these proteins^29,30,34^, the lysozyme binding mode would be predicted to differ from classic MliC/PliC proteins. Furthermore, antibodies to rDn-ACP cross-reacted with rNm-ACP and *vice versa*, which demonstrated the conservation and close relationship with *Neisseria* ACP proteins.

To our knowledge, there are no reports on lysozyme inhibition activity for *Dichelobacter* and our study is the first to show that Dn-ACP is a C-type lysozyme inhibitor. We demonstrated that the rDn-ACP protein prevented lysis of *M. lysodiekticus* cells in the presence of Hewl *in vitro* by inhibiting the enzymatic activity of lysozyme. Comparing the effectiveness of lysis with the rNm-ACP protein, six-fold less rDn-ACP was required and it is interesting to speculate that the strong Hewl inhibition by Dn-ACP may be rooted in the duplication of the ACP domains. Expression of Dn-ACP *in vivo* may enable *Dichelobacter* to avoid a key, ubiquitous vertebrate innate immune defence mechanism, and possibly contribute to establishing bacterial colonization and infection.

Inclusion of rDn-ACP within a defined antigen footrot vaccine or perhaps to existing vaccines including Footvax, might led to the potential induction of ovine antibody responses that inhibit the ability of *Dichelobacter* to avoid lysozyme activity and thus contribute to preventing infection by allowing host innate immune clearance by lysozyme. However, choice of adjuvant is critical for new vaccine(s) that might include rDn-ACP. A variety of different adjuvants were used with both rDn-ACP and rDn-MIP antigens. In general, all of the adjuvants helped stimulate similar high levels of antibodies to both proteins. However, we did observe differences in the ability of rDn-ACP antibodies to inhibit rDn-ACP *in vitro*, which was influenced by the nature of the adjuvant used. Only antisera generated by rDn-ACP-QuilA and rDn-ACP-Al(OH)_3_, and to a lesser extent to rDn-ACP-MONTANIDE and rDn-ACP- Freunds’ mixtures, significantly prevented rDn-ACP protein from inhibiting Hewl activity *in vitro*. It is possible that these adjuvants induce antibodies with higher avidity that bind to the, as yet, uncharacterized rDn-ACP epitopes that are involved in binding to and inhibiting lysozyme. Moreover, despite the fact that immunization with Footvax induced murine antibodies that reacted with rDn-ACP, these antibodies were unable to prevent rDn-ACP inhibition of Hewl activity. Several possible reasons may account for this unexpected lack of antibody function, *e.g*. the oil adjuvant in Footvax is inappropriate; relatively lower levels of antibody were induced, perhaps as a consequence of different molar ratios of recombinant protein and Dn-ACP present in the Footvax dose used for immunization; and/or antibodies induced to Footvax Dn-ACP have lower avidity to functional and/or other non-functional epitopes.

A defined measure of protection would allow comparisons between studies and reduce the need for ovine protection trials, in which sheep would be vaccinated with these antigens in an appropriate adjuvant and the feet then challenged with *D. nodosus*. However, the mechanism of protection against footrot is not clear. Parenteral vaccination with Footvax confers protection, and generating specific interdigital mucosal immune responses may be important. There are *in vitro* assays that provide correlates for protection with other pathogens that express homologue ACP and MIP proteins, *e.g*. the complement-mediated serum bactericidal assays (SBA) described previously for Nm-ACP, Ng-ACP and Nm-MIP^26–28,30,55^ and opsonophagocytosis assays using immortalized murine macrophage cell lines e.g. J774A.1^56^. However, no correlates are available for ovine protection. Nonetheless, we attempted to develop an SBA assay, but a functional bactericidal antibody response induced by antigen compared to sham-immunization could not be quantified, due principally to the sensitivity of *Dichelobacter* to animal sera used as exogenous complement sources. This same issue hindered our attempts to develop an opsonophagocytosis assay using J774A.1 murine macrophages, based on a method described by Romero-Steiner *et al*.^57^. We attempted also a slide agglutination test as originally described by Claxton *et al*.^14^, since previous studies have shown agglutination could be a potential biological marker, *e.g*. the level of serum K-agglutinating antibody stimulated by whole-cell vaccination was associated with protection against footrot^10^. There was visual evidence of partial agglutination of *D. nodosus* bacteria mixed with antisera to Dn-ACP-MONTANIDE, Dn-MIP-(Lip + M) and Dn-MIP-Freund’s in comparison to treatment with NMS, suggesting possible interaction of antibodies to the surface antigen (data not shown). However, this agglutination assay was generally non-discriminatory and relatively insensitive. Recently, a three-dimensional skin explant model was described for studying anaerobic bacterial infections such as footrot^58^, but this has not been developed, and might not be appropriate for, vaccine efficacy testing.

In this study, our rationale for using an inbred mouse species was that it allowed us to pre-screen the antigenicity of both proteins, identify a suitable range of adjuvants and examine the mechanisms of lysozyme inhibition, in a more affordable way. However, the limitation of our study is that mice are maintained in a specific-pathogen free environment and their immune responses may not be similar to those of outbred sheep with immune systems that are exposed to a range of different microbes in their environment. Thus, despite the promising structural/functional features of rDn-ACP and rDn-MIP as putative vaccine antigens, and in the absence of reliable *in vitro* assays for correlates of protection, the vaccine potential of both proteins ultimately requires expensive ovine protection trials.

In summary and to our knowledge, this is the first report of the function of Dn-ACP as a novel lysozyme inhibitor produced by *Dichelobacter*, and increases our knowledge of the distribution of this class of inhibitors amongst human and animal pathogens. A vaccine containing rDn-ACP could contribute to protection by enabling normal lysozyme function and further studies are needed to examine the role of Dn-ACP in particular during infection *in vivo*. Furthermore, MIP proteins have been acknowledged as potential targets for drug therapies during infection^54^ and for vaccine development^25^. Thus, both antigens deserve consideration for next-generation footrot vaccines.

## Methods

### Bacterial strains and growth conditions

*Dichelobacter nodosus* (Beveridge, 1941) ATCC 25549, strain VPI 2340 [11342] (serotype B)^59^ was grown for 3 to 4 weeks on Eugon Agar with defibrinated sheep blood (5% vol/vol) at 37 °C in an anaerobic gas jar with the Anaerogen gas-generating system (Oxoid, United Kingdom). *Escherichia coli* DH5α (cloning) and BL21(DE3) pLysS (protein expression) strains were grown at 37 °C on Luria-Bertani (LB) agar and broth, with or without addition of selective antibiotics and isopropyl-β-1-D-thiogalactopyranoside (IPTG) when necessary.

### Bioinformatics

*D. nodosus* DNO_0012 peptidyl prolyl-cis-trans-isomerase FKBP-type, now DNO_RS00050, and DNO_0725 potential Adhesin Complex Protein, now DNO_RS06795, were used as allele search terms in the https://pubmlst.org/bigsdb?db=pubmlst_dnodosus_isolates database^31^. Isolates number 172 (accessed April, 2019) and amino acid sequences for alleles were produced with Clustal (https://www.ebi.ac.uk/Tools/msa/clustalo/) and dendrograms showing relationships between non-redundant alleles were generated with Jalview 2.8 (http://www.jalview.org/development/release-history/Version-282).

### Sequence alignment and phylogenetic analysis for ACP proteins

Sequences alignment was performed using ClustalX 2.1^32^. Initially, sequences of all ACP were aligned using multiple sequence alignment with maximum iterations and all MliC/PliC were aligned using the same parameters separately. These were each saved as separate alignment profiles. The same method was used to align the N- and C-terminal sequences of Dn-ACP and this was also saved as a profile. Profile alignment was first performed using MliC/PliC and ACP, this alignment was then saved as a separate profile. The Dn-ACP profile was then aligned to the MliC/PliC/ACP profile to produce the final alignment. The aligned sequences were output in a phylogenetic tree format (.ph), which was displayed by FigTree v1.4.3 (http://tree.bio.ed.ac.uk/software/figtree)^60^. Sequence alignments presented were coloured using ESPript (ESPript - http://espript.ibcp.fr)^61,62^. The secondary structure predictions for Dn-ACP sequences were made using Jpred4^63^ and the secondary structure for St-PliC and Ng-ACP were assigned by DSSP (http://swift.cmbi.ru.nl/gv/dssp/)^64^ and based on the crystal structures 3OE3 and 6GQ4 respectively.

### Modelling of Dn-ACP domains

After splitting the sequence of Dn-ACP into two segments based on their similarity, each sequence was loaded into Chimera (http://www.rbvi.ucsf.edu/chimera/)^65^ and a BLAST (https://www.ncbi.nlm.nih.gov/BLAST/) search performed on each sequence. The BLAST search indicated PDB codes 6GQ4 and 5MY7 had sequence similarity. These PDBs were loaded into Chimera and the sequences aligned to Dn-ACP sequences. MODELLER 9.21 (https://salilab.org/modeller/)^66^ was then run locally to generate 250 models of each Dn-ACP sequence (N- and C-term). These were then scored using the web server and the best model saved. Prior to production of figures using PyMol (https://pymol.org/)^67^, the unmodelled lengths of sequences were removed from both models.

### Sequence alignment and modelling of Dn-MIP protein

MIP and other PPIase amino acid sequences were aligned using ClustalX 2.1^32^ - multiple alignment mode, with iterations on every sequence. ESPript software^61,62^ was used to display the alignment. Modelling of Dn-MIP was done as described for Dn-ACP, using *Legionella pneumophila* MIP structure (PDB:1FD9) as the template. PISA software^68^ was used to map the MIP dimerization interface.

### Cloning, expression, and purification of recombinant Dn-ACP and Dn-MIP proteins

DNO_0725 and DNO_0012 were cloned, expressed and purified using methods previously described for rNm-ACP and rNm-MIP proteins, respectively^28,55^. Briefly, the DNO_0725 (*dn-acp*) gene sequence, encoding the amino acid sequence (AA 1–230) for *Dichelobacter nodosus* (Dn)-ACP protein, was amplified by PCR using primers DNO0725 Fw 5′-TTACTCGAGATGAAAAAAGAACAATTTTTAGCGGC-3′ and DNO0725 Rv 5′-TAT AAGCTTTTAACGTGCTTTACACATTTTATAAAGG-3′. The DNO_0012 (*dn-mip*) gene sequence, encoding the amino acid sequence (AA 1–329) for Dn-MIP protein, was amplified by PCR using primers DNO0012 Fw 5′-TAACTCGAGATGATGAAAAAAACTTCTTTAC-3′ and DNO0012 Rv 5′-TATAAGCTTCTATTTTTTAGCCGCTTCATCAGG-3′. The restriction sites for XhoI and HindIII are underlined. Both the 711 bp (*dn-acp*) and the 1008 bp (*dn-mip*) PCR products were purified using a Wizard® SV Gel and PCR Clean-up System (Promega), double digested with XhoI and HindIII restriction enzymes (Promega) at 37 °C for 3 h, and purified from an agarose gel band. The purified digested PCR fragments were then ligated overnight at 4 °C to pRSETA vector, which was previously linearized with both same restriction enzymes. The recombinant vectors (pRSETA-*dn-acp* and pRSETA-*dn-mip*), encoding for Dn-ACP and Dn-MIP proteins fused to a N-terminal hexa-histidine tag, were transformed into *E.coli* DH5α competent cells for plasmid amplification and subsequently into competent *E. coli* BL21 (DE3) pLysS cells for protein expression. Recombinant (r)Dn-ACP protein was purified by Ni-NTA affinity chromatography under denaturing conditions. Bound protein was eluted using 100 mM NaH_2_PO_4_, 10 mM Tris-HCl, 6 M GuHCl and 250 mM imidazole buffer, pH 8.0, precipitated with 5% (v/v) Trichloroacetic acid (TCA), and subsequently resuspended in phosphate buffered saline (PBS), pH 7.4 with 0.5% (w/v) sodium dodecyl sulphate (SDS) for solubilization. rDn-MIP protein was purified by Ni-NTA affinity chromatography under non-denaturing conditions. Bound protein was eluted using 50 mM NaH_2_PO_4_, 300 mM NaCl and 250 mM imidazole buffer, pH 8.0, and subsequently dialyzed against PBS, pH 7.4 for 48 h. Protein concentration was determined using the BCA^TM^ Protein Assay Kit (Pierce). The molecular mass (*Mr*) of rDn-ACP and rDn-MIP proteins, with a 39 AA-long N-terminal hexa-histidine tag (MRGSHHHHHHGMASMTGGQQMGRDLYDDDDKDRWGSELE), was 30.2 kDa and 40.1 kDa, respectively, as shown by SDS-PAGE (Supplementary Fig. 8). The purified recombinant proteins were stored at −20 °C until needed.

### Immunization of mice with rDn-ACP and rDn-MIP proteins

BALB/c mice (H-2^d^ haplotype) were bred within the animal facilities of the university under standard conditions of temperature and humidity with a 12 h lighting cycle and with food and water available *ad libitum*. Groups of five BALB/c mice of approximate equal sizes and weights (6–7 weeks of age) were immunized with purified rDn-ACP or rDn-MIP protein prepared separately with the following adjuvants.

rDn-ACP and rDn-MIP stock solutions were diluted in sterile saline and were adsorbed to **(i) AL(OH)**_**3**_, **(ii) incorporated into Liposomes**, **(iii) Liposomes plus monophosphoryl lipid A (MPLA)**, **(iv) Zwitterion (Zw) detergent 3–14** and **(v) Zw 3–14 plus MPLA**, as described previously^30^. Control preparations without protein were prepared similarly.

**(vi) Freund’s adjuvant**. For the primary immunization, a volume of 0.35 mL of a solution of 400 µg/mL of rDn-ACP or rDn-MIP in sterile saline was mixed with 0.35 mL of Freund’s Complete Adjuvant (FCA) and an emulsion prepared by repeated passage through a 21G syringe needle. The second and third doses were prepared similarly in Freund’s Incomplete Adjuvant (FIA). Control FCA and FIA preparations were prepared similarly without protein.

**(vii) Quil A** (Sigma). A solution of 1 mg/mL Quil A was prepared in saline and sterilized by filtration through a 0.22 µm filter. A volume of 0.105 mL of sterile Quil A (1 mg/mL) was added to 0.35 mL of a solution of 400 µg/mL of rDn-ACP or rDn-MIP in sterile saline and the volume adjusted to 0.7 mL with sterile saline. Control Quil A preparation was prepared similarly without protein.

**(viii) MONTANIDE ISA 71 VG** (Batch T35031). This oil adjuvant was kindly provided by Seppic (France) for the production of water-in-oil emulsions. MONTANIDE was prepared in a 70:30 ratio with protein in saline, following the manufacturer’s recommendations. A volume of 0.49 mL of MONTANIDE was mixed with 0.21 mL of saline containing 140 µg of rDn-ACP or rDn-MIP in sterile saline and an emulsion prepared as described for Freund’s adjuvants. Control MONTANIDE emulsion was prepared with 0.49 mL of adjuvant with 0.21 mL of saline alone.

Each mouse was immunized with a volume of 100 µL containing 20 µg of protein and adjuvant per dose on days 0, 14 and 28. Control mice received 100 µL of adjuvant alone. One group (n = 5) was kept as a source of normal mouse serum (NMS).

**Immunization of mice with (ix) Footvax** (MSD Animal Health). Footvax contains per 1 mL dose, 10 µg of pili of each of *D. nodosus* serotypes A, B1, B2, C, D, E, F, G and H and 5 × 10^8^ cells of *D. nodosus* serotype I (New Zealand strain), delivered in 60% light mineral oil NF and 4.5% manide oleate as adjuvants, and 0.015% thiomersal as a preservative. Each mouse was immunized with 100 µL of Footvax on days 0, 14, and 28.

All mice were terminally bled by cardiac puncture under anaesthesia on day 42. All sera were stored at −20 °C until required and were decomplemented by heating at 56 °C for 30 min where stated.

### Ethics Statement

This study complied with the animal experimentation guidelines of the Home Office (HO), with approval granted under the Animals (Scientific Procedures Act, 1986) with a HO project licence number PPL 30/3126. The study was approved by the Animal Welfare and Ethics Review Board (AWERB) at the authors’ institution (University of Southampton, no number assigned). Animal health and welfare was assessed daily by qualified animal technicians and no animals suffered significant adverse effects.

### Characterization of biological and functional properties of antibodies to rDn-ACP and rDn-MIP. (i) Recombinant protein ELISA

Individual murine antisera were reacted in ELISA against rDn-ACP and rDn-MIP proteins (0.1 µg protein/well), as described previously^69^. Briefly, flat-bottomed polystyrene microtitre plates were coated overnight at 37 °C with recombinant protein in 0.05 M sodium carbonate buffer, pH 9.6. Wells were washed with PBS containing Tween-20 (0.05% v/v, PBST) and antisera were diluted serially in PBST containing 1% (w/v) BSA and the plates incubated for 1 h at 37 °C. After washing, antibody binding was detected using the appropriate HRP conjugate with 3,3′,5,5′-tetramethylbenzidine and H_2_0_2_ as enzyme substrate. Absorbance was measured at λ450 nm after 10 min of incubation with enzyme substrate, and the ELISA titre, extrapolated from the linear portion of the serum titration curve, was taken as the reciprocal dilution which gave an increase in absorbance of 0.1 U after 10 min.

**(ii) Whole cell ELISA**. The wells of flat-bottomed polystyrene microtitre plates were coated with 1 µg of live *D. nodosus* cells/well in buffer carbonate buffer, pH 9.6, overnight at 4 °C. For this assay, individual antisera were tested at a single dilution of 1/100 on whole cells, which was the lowest dilution determined as optimal for avoiding non-specific reactivity of control, sham sera or normal mouse serum. Washing steps and antibody binding detection were done as described above. Absorbance measurements at λ450 nm were made after 10 min of incubation with enzyme substrate, as described above. For the recombinant protein and whole cell ELISA, data were compared using a two-sample *t*-Test, with P values < 0.05 considered significant.

**(iii) Western immunoblotting**. A *D. nodosus* whole cell lysate preparation (15 µg/well and 50 µg/well for testing antisera to rDn-MIP and antisera rDn-ACP antisera, respectively) and purified recombinant proteins (1 μg each), were separated on SDS-PAGE and then transferred to nitrocellulose by semi-dry blotting. After incubation with pooled murine sera (1/100 dilution; n = 5 animals), immunological reactivity was detected by using anti-mouse immunoglobulin-alkaline phosphatase conjugate (Bio-Rad, UK) as described previously^69^.

**(iv) Hen egg-white lysozyme (Hewl) inhibition assay**. Lysis kinetics of freeze-dried *M. lysodeikticus* cells (ATCC4698 – Sigma Aldrich) suspended at 1 mg/mL in 10 mM potassium phosphate buffer (PPB) pH 7.0 supplemented with a protease inhibition cocktail (Roche) were monitored over time (every 5 min for 2 h and a final end point of 24 h incubation) by measuring the change in optical density (OD) at λ_595_nm in the presence or absence of 10 U/well of Hen egg-white lysozyme (Hewl) at 25 °C. rDn-ACP protein inhibition activity of Hewl was determined by dose response (3–90 pmol/well) experiments, for which r*Neisseria meningitidis* (Nm)-ACP (6–180 pmol/well) and rDn-MIP (60–180 pmol/well) proteins were tested as positive and negative controls for Hewl inhibition, respectively. Possible detrimental effect of SDS on the integrity of *M. lysodeikticus* cells and/or on the enzymatic activity of Hewl was tested by adding the same final concentration of SDS (2.25 × 10^–3^% (w/v)) to a suspension of the bacteria with or without Hewl and compared their kinetic curves with the same samples without SDS. Adjuvant effect of rDn-ACP antisera on preventing rDn-ACP protein from inhibiting Hewl was demonstrated by adding to the reaction (for the condition of 30 pmol/well of rDn-ACP only) a 1/16 dilution of pooled (n = 5 mice per group), decomplemented murine antisera raised against the recombinant protein delivered in the different formulations, as well as the corresponding sham sera, Footvax antisera and NMS.

For testing if Hewl antimicrobial activity was responsible for *M.lysodiekticus* cell death, Hewl was boiled for 1 h and lysis kinetic experiments were done using the same conditions described above, comparing boiled Hewl with untreated Hewl and control (no Hewl).

## Supplementary information

Supplementary Information

## Data Availability

All data generated or analysed during this study are included in this published article (and its Supplementary Information files).
