# Supplementary material for: Characterization of two putative Dichelobacter nodosus footrot vaccine antigens identifies the first lysozyme inhibitor in the genus
Source: Sci Rep. 2019 Jul 11;9:10055. doi: 10.1038/s41598-019-46506-z (PMC6624275; doi:10.1038/s41598-019-46506-z)
Supplement: Supplementary file 1 — Supplementary Information [file 41598_2019_46506_MOESM1_ESM.pdf]

Characterization of two putative *Dichelobacter nodosus* footrot vaccine antigens identifies the first lysozyme inhibitor in the genus

Maria Victoria Humbert[a], Alexandra Jackson[a], Christian M. Orr[b], Ivo Tews[c], Myron Christodoulides[a*]

[a]Neisseria Research Group, Molecular Microbiology, Academic Unit of Clinical and Experimental Sciences, Sir Henry Wellcome Laboratories, University of Southampton Faculty of Medicine, Southampton, United Kingdom.

[b]Beamline I23, Diamond Light Source, Harwell Science and Innovation Campus, Didcot, Oxfordshire, United Kingdom.

[c]Biological Sciences, Institute for Life Sciences, B85 Highfield Campus, University of Southampton, Southampton, United Kingdom.

*Address correspondence to Myron Christodoulides, mc4@soton.ac.uk

Supplementary Table 1. Analysis of DNO_0725/DNO_RS06795 (*dn-acp*) allele diversity and frequency of *D. nodosus* isolates collated from the PubMLST (https://pubmlst.org/dnodosus/) database.

Non-redundant alleles	No of isolates
1(+3,4,7,8,9,10,11,14,15,16)	146
2(+17)	14
5	6
6(+12, 13)	4
**Total**	**170**

Database accessed in April 2019 displayed 17 allelic loci generating 4 non-redundant proteins, within a total population of 170 identified isolates, with 2 isolates with no allele defined. Numbers in parentheses indicate that the alleles produce proteins with identical amino acid sequences.

Supplementary Table 2. Analysis of DNO_0012/ DNO_RS00050 (*dn-mip*) allele diversity and frequency of *D. nodosus* isolates collated from the PubMLST (https://pubmlst.org/dnodosus/) database.

Non-redundant alleles	No of isolates
1 (+2, 11)	83
3(+5,16,24)	14
4(+6,9,12,13,14,15,17,18,19,21,25)	62
7	1
8	1
10	1
20	1
22	2
23	1
**Total**	**166**

Database accessed in April 2019 displayed 25 allelic loci generating 9 non-redundant proteins, within a total population of 166 identified isolates, with 6 isolates without defined allele. Numbers in parentheses indicate that the alleles produce proteins with identical amino acid sequences.

Supplementary Fig. 1. ***In silico*** **analysis of DNO_0725/DNO_RS06795 (*dn-acp*) allele diversity and frequency from all *D. nodosus* isolates in the PubMLST (https://pubmlst.org/dnodosus/) database.** Database was accessed in April 2019. **A)** Dendrogram showing the clustering of non-redundant Dn-ACP proteins. The dendrogram was assembled with Jalview 2.9 (www.jalview.org). **B)** Alignment of non-redundant DNO_0725/DNO_RS06795 amino acid sequences for all *D. nodosus* isolates in the PubMLST database. Amino acid sequence alignment was generated using Clustal Omega (http://www.ebi.ac.uk/Tools/msa/clustalo/). A denotes Allele. Asterisk (*****) denotes fully conserved amino acid residue; colon (**:**) indicates conservation between groups of strongly similar properties; period (**.**) denotes conservation between groups of weakly similar properties.

Supplementary Fig. 2. ***In silico*** **analysis of DNO_0012/ DNO_RS00050 (*dn-mip*) allele diversity and frequency from all *D. nodosus* isolates in the PubMLST (https://pubmlst.org/dnodosus/) database.** Database was accessed in April 2019. **A)** Dendrogram showing the clustering of non-redundant Dn-MIP proteins. The dendrogram was assembled with Jalview 2.9 (www.jalview.org). **B)** Alignment of non-redundant DNO_0012/ DNO_RS00050 amino acid sequences for all *D. nodosus* isolates in the PubMLST database. Amino acid sequence alignment was generated using Clustal Omega (http://www.ebi.ac.uk/Tools/msa/clustalo/). A denotes Allele. Asterisk (*****) denotes fully conserved amino acid residue; colon (**:**) indicates conservation between groups of strongly similar properties; period (**.**) denotes conservation between groups of weakly similar properties.

Supplementary Fig. 3. Cross-reactivity of antisera to rDn-ACP and to rNm-ACP proteins. Pooled mice antisera (1/100 dilution; n = 5 animals) raised against **A)** rDn-ACP or **B)** rNm-ACP protein (29) delivered in $Al(OH)_3$ was reacted in Western blot against the

corresponding purified recombinant immunizing antigen and the homologous protein (1 μg each). rDn-ACP protein was recognised as a single band of *Mr* ~30.2 kDa and rNm-ACP protein as a single band of 17.8 kDa (identified by the arrow), with antisera raised against both the *Dichelobacter* and the meningococcal antigen. Samples were processed in parallel on the same western blots and representative full-length western blots are shown (of $n \geq 3$ experiments for each blot). Images were captured on a HP ScanJet flatbed scanner.

Supplementary Fig. 4. Alignment of MIP proteins. Alignments were prepared with ClustalX 2.1. and sequences shown from top to bottom are *Vibrio cholerae* Peptidyl-prolyl cis-trans isomerase (UniProt:Q9KP11), *Escherichia coli* FKBP-type peptidyl-prolyl cis-trans isomerase (UniProt:P0A9L3), *Legionella pneumophila* outer membrane protein MIP (UniProt:Q70YI1) as well as its structure (PDB:1FD9) that was used as a template for the homology model of Dn-MIP, the Dn-MIP investigated in this study, the *Trypanosoma cruzi* MIP protein (UniProt:Q09734) as well as its structure (PDB:1JVW), the sequence of the *Homo sapiens* FK506 binding protein (R42K and H87V exchange variant) in complex with the inhibitor, shown in the comparison in Fig 2 (PDB:1BKF), and the sequence of the *Escherichia coli* FkpA structure (PDB:1Q6U). ESPript was used to prepare the figure.

Supplementary Fig. 5. Kinetics of purified rDn-ACP protein inhibitory activity of Hen egg-white lysozyme (Hewl) *in vitro*. Lysis of a 1 mg/ml *Micrococcus lysodeikticus* cell suspension (expressed as a reduction in $OD\lambda_{595}$nm against time) in the absence or in the presence of increasing concentrations of **A)** rDn-ACP (3 – 90 pmol/well), **B)** rNm-ACP (6 – 180 pmol/well) or **C)** rDn-MIP (60 – 180 pmol/well), and 10 U/well of Hewl. The curves represent the mean absorbance ($OD\lambda_{595}$nm) and the error bars represent the corresponding standard error of the mean (SEM) of three independent experiments. Data were compared with a paired *t*-Test and the asterisks (*****) denote significant difference ($P<0.05$) in $OD\lambda_{595}$nm in comparison to the control treatment with Hewl only. The presence of SDS did not affect

the integrity of the bacterial cells nor the lytic enzymatic activity of Hewl in the conditions tested with insoluble rDn-ACP and rNm-ACP proteins (rDn-MIP is a soluble protein and therefore do not require SDS for solubilisation). The addition of the recombinant proteins in the absence of Hewl did not induce lysis on *M. lysodeikticus* cells.

Supplementary Fig. 6. ***M. lysodiekticus*** **cell lysis in the presence of Hewl is due to the enzyme´s lytic function only and not to its antimicrobial activity.** Bacterial suspensions (1 mg/mL) were treated with native Hewl (10 U/well), boiled Hewl (10 U/well) or left untreated (control), and absorbance measured at intervals of 5 min over 2 h. The kinetic curves represent the mean absorbance values ($OD_{\lambda 595}$nm) and the error bars represents the SEM of n = 3 independent experiments. Data were compared with a paired *t*-Test.

Supplementary Fig 7. Kinetics of inhibition of rDn-ACP mediated by antibodies against the protein, and effect of the adjuvant on the efficiency of antibodies to inhibit rDn-ACP. Hewl inhibitory activity by rDn-ACP (30 pmol/well) was analysed as a reduction in $OD_{\lambda 595}$nm against time of a *M. lysodeikticus* cell suspension (1 mg/ml) in the presence or absence of pooled decomplemented murine antisera (n = 5 mice per group) raised against rDn-ACP protein in different formulations and sera from sham immunised mice for each of the adjuvants tested. The symbols represent the mean absorbance ($OD_{\lambda 595}$nm) from n = 3 independent experiments and the error bars represent the corresponding SEM. Antisera to rDn-ACP (delivered in QuilA, $Al(OH)_3$, Freund’s, Montanide and Zw 3-14 + MPLA) with significant inhibitory effect on rDn-ACP enzymatic activity are marked with an asterisk (*****). Antisera to rDn-ACP (delivered in Zw 3-14, Liposomes and Liposomes + MPLA), all sham immunized antisera (all adjuvants tested), antisera to Footvax and NMS had no significant effect on rDn-ACP inhibitory activity of lysozyme and are all grouped as ‘(1)’.

Supplementary Fig. 8. SDS PAGE gels of purified recombinant Dn-ACP and Dn-MIP proteins. Recombinant **(**r)Dn-ACP protein (*Mr* ~30,200, identified by the arrow) was

expressed as an insoluble recombinant protein and purified under denaturing conditions. rDn-MIP protein (*Mr* ~40,1000, identified by the arrow) was expressed as soluble recombinant protein and purified under native conditions. Samples were run in parallel on the same SDS-PAGE gels and a representative full-length gel is shown (of n = 3 experiments). Images were captured on a HP ScanJet flatbed scanner.

A)

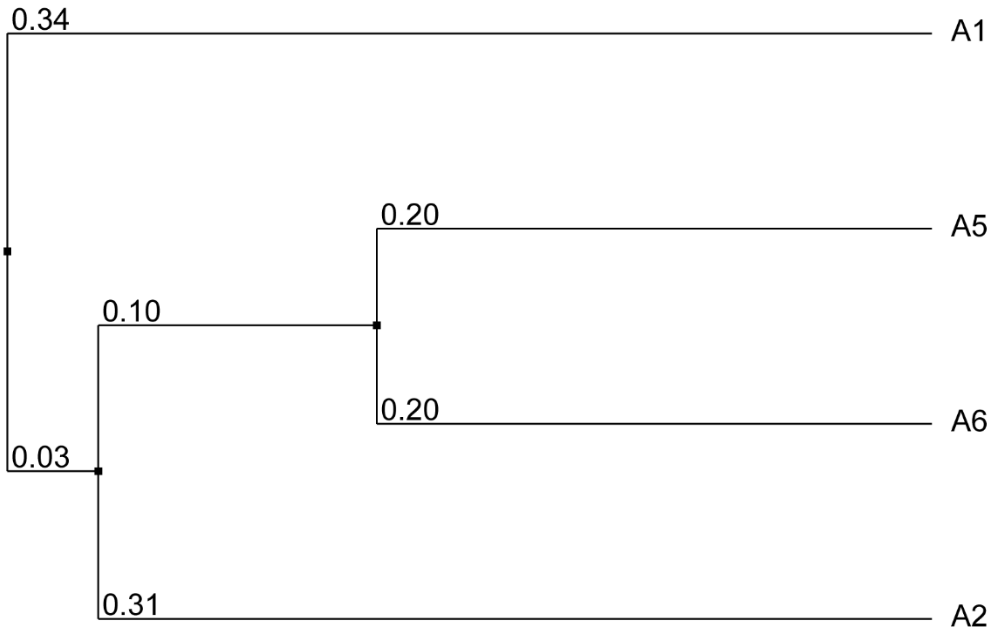

B)

```
A5        MKKEQFLAAALLVFGFSTAAHAELMSYRCDQDRKIDVVYSFNSAGVPTKAEVMIQGKKRV 60
A2        MKKEQFLAAALLVFGFSTAAHAELMSYRCDQDRKIDVVYSFNSAGVPTKAEVMIQGKKRV 60
A1        MKKEQFLAAALLVFGFSTAAHAELMSYRCDQDRKIDVVYSFNSAGVPTKAEVMIQGKKRV 60
A6        MKKEQFLAAALLVFGFSTAAHAELMSYRCDQDRKIDVVYSFNSAGVPTKAEVMIQGKKRV 60
          ************************************************************

A5        LNYNMDRSDNVGSFFNDQAGYELSSNIITRDDYHSSSIMIMSPKSEILFKNCSAAMSTIQ 120
A2        LNYNMDRSDNVGSFFNDKAGYELSSNIITRDDYHSSSIMIMSPKSEILFKNCSAAMSTIQ 120
A1        LNYNMDRSDNVDSFFNDKAGYELSSNIITRDDYHSSSIMIMSPKSEILFKNCSAAMSTIQ 120
A6        LNYNMDRSDNVGSFFNDKAGYELSSNIITRDDYHSSSIMIMSPKSEILFKNCSAAMSTIQ 120
          ***********.*****:******************************************

A5        PVMSEDAPSVDANVVTSVKQANYVCQQGKKMKVEYGFNEVGIPVYALVNFKGTKLKLPYD 180
A2        PVMSEDAPSVDANVVTSVKQANYICQQGKKMKVEYGFNEVGIPVYALVNFKGTKLKLPYD 180
A1        PVMSEDAPSVDANVVTSVKQANYVCQQGKKMKVEYGFNEVGIPVYALVNFKGTKLKLPYD 180
A6        PVMSEDAPSVDANVVTSVKQANYVCQQGKKMKVEYGFNEVGIPVYALVNFKGTKLKLPYD 180
          ***********************:************************************

A5        LDQSSEASTFFTKDGYVFAGDGMTVDTYRNSFMMLTAPSNEILYKMCKAR   230
A2        LDQSSEASTFFTKDGYVFAGDGMTVDTYRNSFMMLTAPSNEILYKMCKAR   230
A1        LDQSSEASTFFTKDGYVFAGDGMTVDTYRNSFMMLTAPSNEILYKMCKAR   230
A6        LDQSSEASTFFTKDGYVFAGDGMTVDTYRNSFMMLTAPSNEILYKMCKAR   230
          **************************************************
```

Supplementary Figure 1

A)

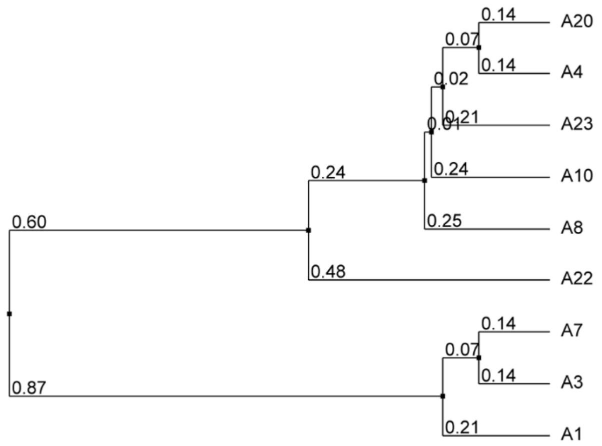

B)

```
A7       MKKTSLLLSAAIALSLTQVYAGVVLKNEGQKVGYAIGVDMGSSIAQLGISDGEELDFNAV 60
A20      MKKTSLLLSAAIALSLTQVYAGVVLKNEGQKVGYAIGVDMGSSIAQLGISDGEELDFNAV 60
A22      MKKTSLLLSAAIALSLTQVYAGVVLKNEGQKVGYAIGVDMGSSIAQLGISDGEELDFNAV 60
A23      MKKTSLLLSAAIALSLTQVYAGVVLKNEGQKVGYAIGVDMGSSIAQLGISDGEELDFNAV 60
A10      MKKTSLLLSAAIALSLTQVYAGVMLKNEGQKVGYAIGVDMGSSIAQLGISDGEELDFNAV 60
A8       MKKTSLLLSAAIALSLTQVYAGVVLKNEGQKVGYAIGVDMGSSIAQLGISDGEELDFNAV 60
A1       MKKTSLLLSAAIALSLTQVYAGVVLKNEGQKVGYAIGVDMGSSIAQLGISDGEELDFNAV 60
A3       MKKTSLLLSAAIALSLTQVYAGVVLKNEGQKVGYAIGVDMGSSIAQLGISDGEELDFNAV 60
A4       MKKTSLLLSAAIALSLTQVYAGVVLKNEGQKVGYAIGVDMGSSIAQLGISDGEELDFNAV 60
         ***********************:*************************************

A7       ILGLRDAYQKKDLLLTQDEMTKTLQDFSEKRLQAMKKEMEKIAAEEAGKGKAFLEENAKK 120
A20      ILGLRDAYQKKDLLLTQDEMTKTLQDFSEKRLQAMKKEMEKIAAEEAGKGKAFLEENAKK 120
A22      ILGLRDAYQKKDLLLTQDEMTKTLQDFSEKRLQAMKKEMEKIAAEEAEKGKAFLEENAKK 120
A23      ILGLRDAYQKKDLLLTQDEMTKTLQDFSEKRLQAMKKEMEKIAAEEAEKGKAFLEENAKK 120
A10      ILGLRDAYQKKDLLLTQDEMTKTLQDFSEKRLQAMKKEMEKIAAEEAEKGKAFLEENAKK 120
A8       ILGLRDAYQKKDLLLTQDEMTKTLQDFSEKRLQAMKKEMEKIAAEEAEKGKAFLEENAKK 120
A1       ILGLRDAYQKKDLLLTQDEMTKTLQDFSEKRLQAMKKEMEKIAAEEAEKGKAFLEENAKK 120
A3       ILGLRDAYQKKDLLLTQDEMTKTLQDFSEKRLQAMKKEMEKIAAEEAEKGKAFLEENAKK 120
A4       ILGLRDAYQKKDLLLTQDEMTKTLQDFSEKRLQAMKKEMEKIAAEEAEKGKAFLEENAKK 120
         *********************************************** ************

A7       DGVITTESGLQYKVVKKGTGAKPNSDDRVTVDYTGTLIDGTEFDSSKGREPITFNVQDVI 180
A20      DGVITTESGLQYKVVKKGTGAKPNSDDRVTVDYTGTLIDGTEFDSSKGREPITFNVQDVI 180
A22      DGVITTESGLQYKVVKKGTGAKPNSDDRVTVDYTGTLIDGTEFDSSKGREPITINVQDVI 180
A23      DGVITTESGLQYEVVKKGTGAKPNSDDRVTVDYTGTLIDGTEFDSSKGREPITFNVQDVI 180
A10      DGVITTESGLQYKVVKKGTGAKPNSDDRVTVDYTGTLIDGTEFDSSKGREPITFNVQDVI 180
A8       DGVITTESGLQYKVVKKGTGAKPNSDDRVTVDYTGTLIDGTEFDSSKGREPITFNVQDVI 180
A1       DGVITTESGLQYKVVKKGTGAKPNSDDRVTVDYTGTLIDGTEFDSSKGREPITINVQDVI 180
A3       DGVITTESGLQYKVVKKGTGAKPNSDDRVTVDYTGTLIDGTEFDSSKGREPITFNVQDVI 180
A4       DGVITTESGLQYKVVKKGTGAKPNSDDRVTVDYTGTLIDGTEFDSSKGREPITFNVQDVI 180
         ************:****************************************:******

A7       AGWVEGLQLMTEGANYIFYIPSDLAYGSRGAGNAIPPNATLIFDVNLLKIEKNEAEAEAD 240
A20      AGWVEGLQLMTEGANYIFYIPSDLAYGSRGAGNAIPPNATLIFDVNLLKIEKNEA--EAD 238
A22      AGWVEGLQLMTEGANYIFYIPSDLAYGSRGAGNAIPPNATLIFDVNLLKIEKNEAE--AD 238
A23      AGWVEGLQLMTEGANYIFYIPSDLAYGSRGAGNAIPPNATLIFDVNLLKIEKNEAE--AD 238
A10      AGWVEGLQLMTEGANYIFYIPSDLAYGSRGAGNAIPPNATLIFDVNLLKIEKNEAE--AD 238
A8       AGWVEGLQLMTEGANYIFYIPSDLAYGSRGAGNAIPPNATLIFDVNLLKIEKNEAE--AD 238
A1       AGWVEGLQLMTEGANYIFYIPSDLAYGSRGAGNAIPPNATLIFDVNLLKIEKNEAEAEAD 240
A3       AGWVEGLQLMTEGANYIFYIPSDLAYGSRGAGNAIPPNATLIFDVNLLKIEKNEAEAEAD 240
A4       AGWVEGLQLMTEGANYIFYIPSDLAYGSRGAGNAIPPNATLIFDVNLLKIEKNEA--EAD 238
         *******************************************************   **

A7       KKESIAKSINKSLEEATEIVKAEVEADKKESIAKSINKSLEEATETVKAEAEADKKEAIA 300
A20      KKESIAKSINKSLEEATEIVKAEVEADKKESIAKSINKSLEEATETVKAEAEADKKEAIA 298
A22      KEESIAKSINKSLEGATEIVKAEVEADKKESIAKSINKSLEEATETVKAEAEADKKEAIA 298
A23      KKESIAKSINKSLEEATEIVKAEVEADKKESIAKSINKSLEEATETVKAEAEADKKEAIA 298
A10      KKESIAKSINKSLEEATEIVKAEVEADKKESIAKSINKSLEEATETVKAEAEADKKEAIA 298
A8       KEESIAKSINKSLEEATEIVKAEVEADKKESIAKSINKSLEEATETVKAEAEADKKEAIA 298
A1       KKESIAKSINKSLEEATEIVKAEVEADKKESIAKSINKSLEEATETVKAEAEADKKEAIA 300
A3       KKESIAKSINKSLEEATEIVKAEVEADKKESIAKSINKSLEEATETVKAEAEADKKEAIA 300
A4       KKESIAKSINKSLEEATEIVKAEVEADKKESIAKSINKSLEEATETVKAEAEADKKEAIA 298
         *:************ *********************************************

A7       NSINKSLEEAAEAVKEVIEAKPDEAAKK  328
A20      NSINKSLEEAAEAVKEVIEAKPDEAAKK  326
A22      NSINKSLEEAAEAVKEVIEAKPDEAAKK  326
A23      NSINKSLEEAAEAVKEVIEAKPDEAAKK  326
A10      NSINKSLEEAAEAVKEVIEAKPDEAAKK  326
A8       NSINKSLEEAAEAVKEVIEAKPDEAAKK  326
A1       NSINKSLEEAAEAVKEVIEAKPDEAAKK  328
A3       NSINKSLEEAAEAVKEVIEAKPDEAAKK  328
A4       NSINKSLEEAAEAVKEVIEAKPDEAAKK  326
         ****************************
```

Supplementary Figure 2

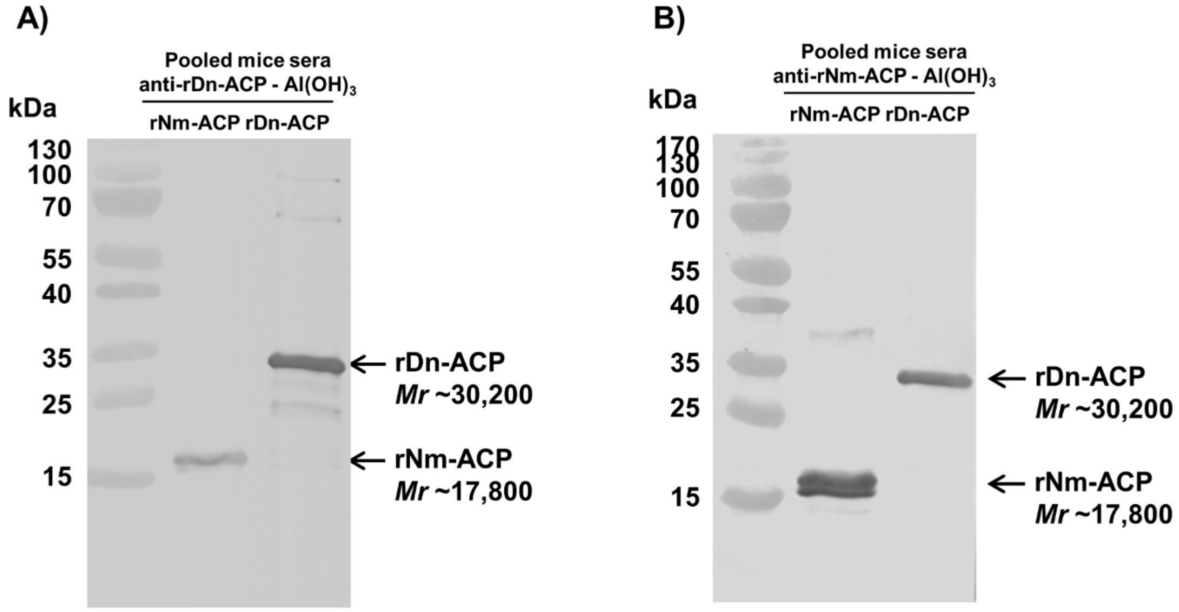

Supplementary Figure 3

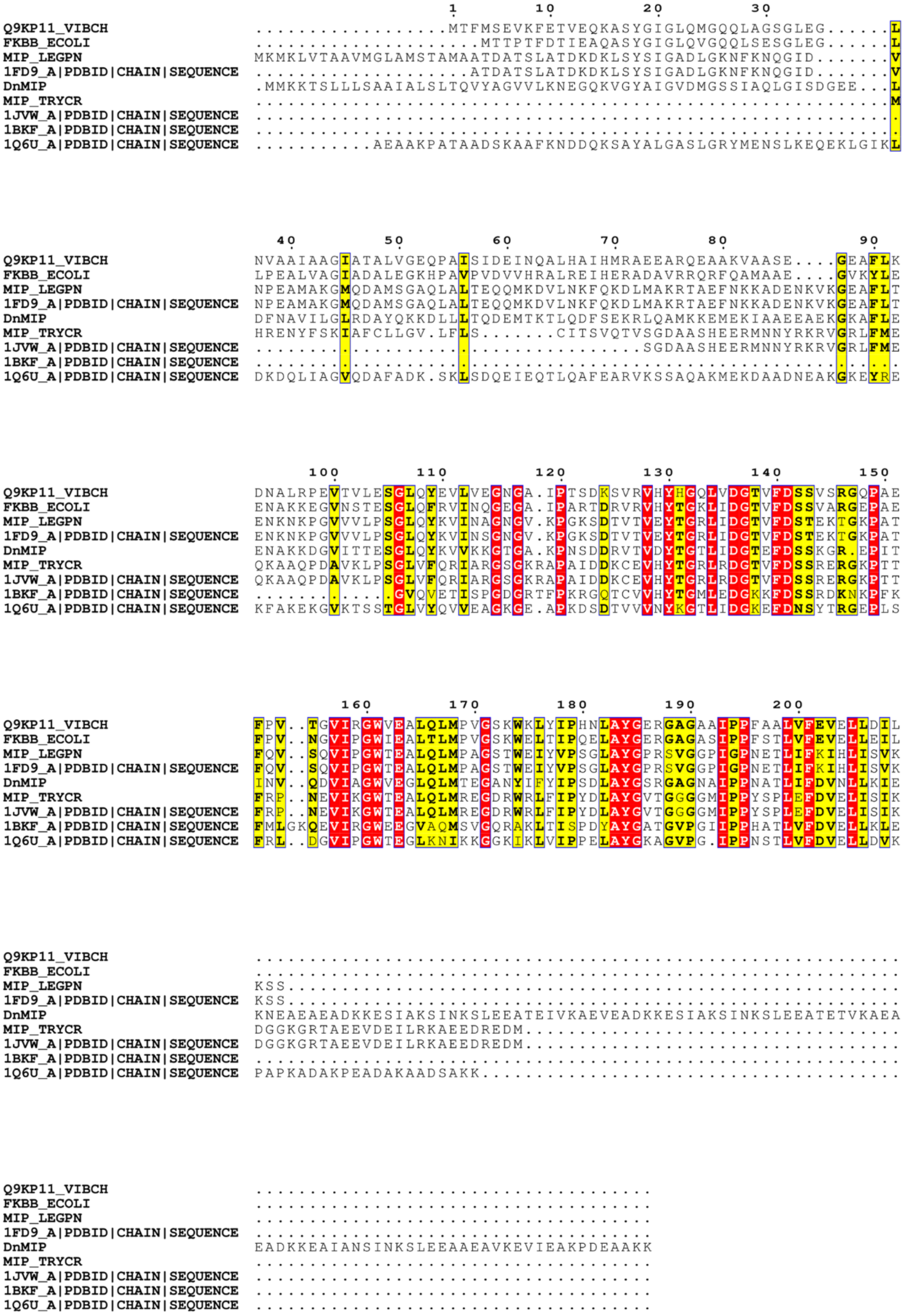

Supplementary Figure 4

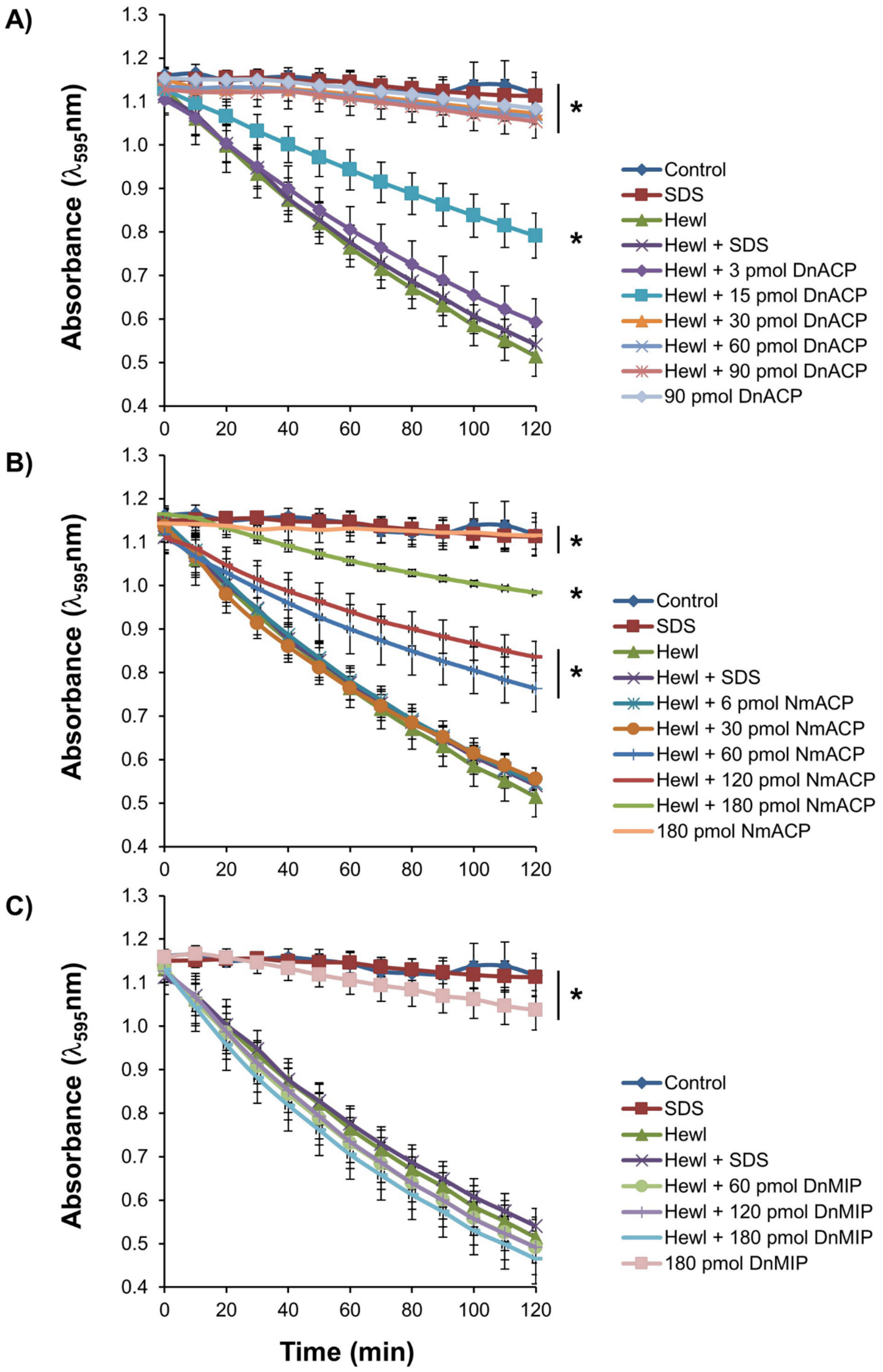

Supplementary Figure 5

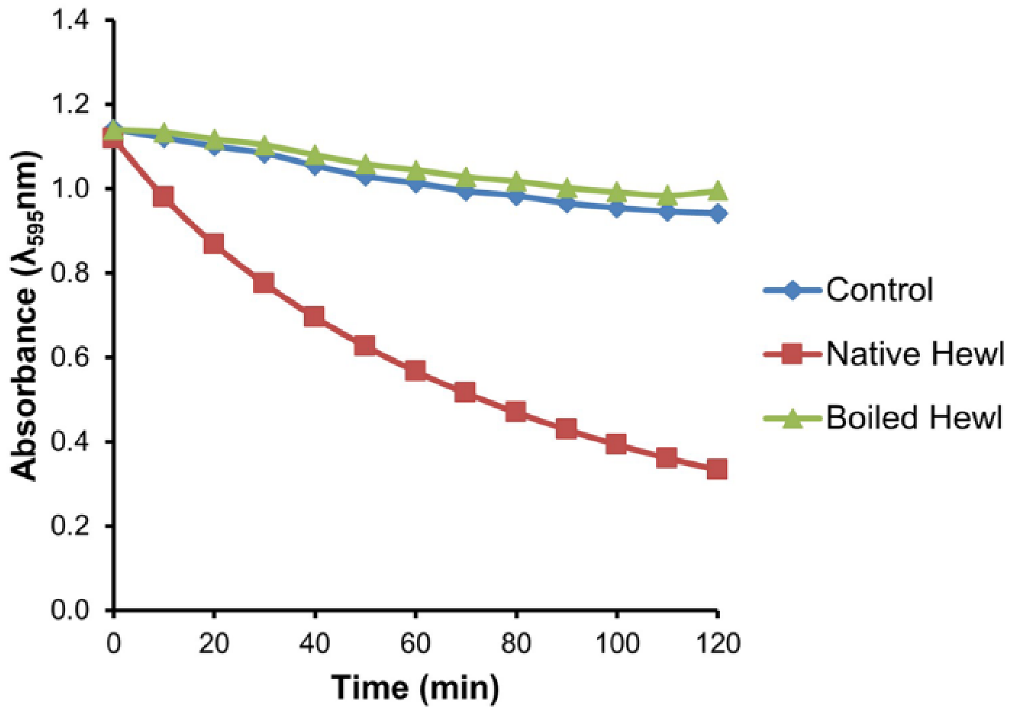

Supplementary Figure 6

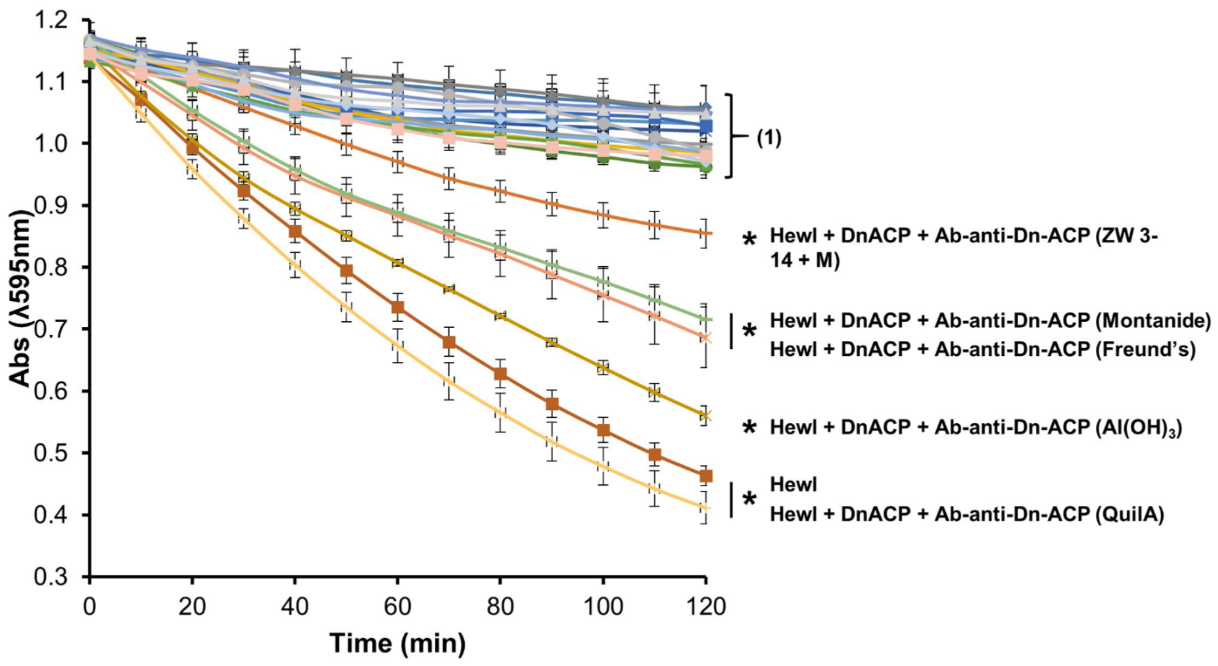

Supplementary Figure 7

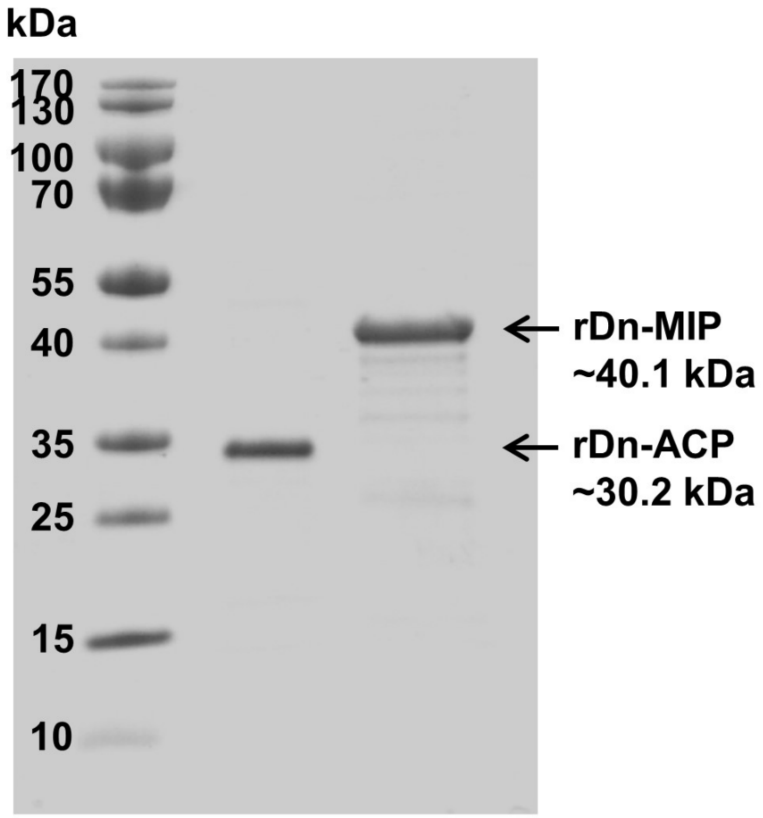

Supplementary Figure 8
